# Analysing of the sustainable development goals in Damascus University during Syrian crisis using the strategy in the university and the bibliometrics data from SciVal

**DOI:** 10.1007/s43621-023-00140-y

**Published:** 2023-05-22

**Authors:** Marwan Al-Raeei

**Affiliations:** Damascus University, Damascus, Syrian Arab Republic

**Keywords:** Developing country, Sustainability, Sustainable development goals, Damascus University, Future

## Abstract

Most countries strive to reach effective sustainable development policies, given the repercussions of this policy on many aspects, such as the economic growth of countries. The adoption of policies of sustainability by developing countries may lead to their development faster than expected. This research aims to study the strategies applied in one of the universities of developing countries and the sustainability policies adopted at that university which is Damascus University. The study focuses on the time during last four years of the Syrian crisis through several factors, with a focus on the analysis based on the SciVal and Scopus databases and the strategies applied by the university itself. In this research, we use the method of extracting and analysing the data of the sixteen sustainable development goals (SDGs) of Damascus University within Scopus and SciVal. We also analyse the strategies used in the university in order to reach some of the determinants of SDGs. By analysing Scopus and SciVal data, we find that the third goal of SDGs is the most widespread in terms of scientific research in Damascus University. We find that the application of such policies led to an important goal in the environment, which is the ratio of green space in Damascus University reaching to more than 63 percent of the total flat area of the university. In addition, we find that the application of sustainable development policies led to the generation of energy from renewable sources by 11% of the total electrical energy consumed at the university. The university has been able to reach lots of indicators of the sustainable development goals and it remains apply others.

## Introduction

The policy of sustainable development is an important factor in the development of different countries around the world. A large number of countries in the world have begun to respond to the sustainability goals that were launched in 2015. In that year, the United Nations, through its General Assembly, launched the Global Goals GGs or the Sustainable Development Goals SDGs. These goals included seventeen different goals that focus on sustainable future development over all parts of the world. The first goal of the SDGs aims for the ending of poverty in all countries of the world, and this goal is defined as "No Poverty" goal [1]. The first SDGs goal is known as SDG-1 or GG-1. The second goal of the SDGs aims for the ending of the hungry in all societies over the world, and this goal is defined as "Zero Hunger" goal [2]. The second SDGs goal is known as SDG-2 or GG-2. As we note, the first and second goals of sustainable development are linked to each other. The goal which focuses on the human health is the third goal of the SDGs goals, which aims at a dignified and healthy life for all individuals of all ages worldwide. The third SDGs goal is known as SDG-3 or GG-3, and this goal is defined as "Well Being" and "Good Health" [3]. The goal which focuses on the education is the fourth goal of the SDGs [4], where the fourth goal of the SDGs aims for securing the education for all. The fourth goal of the SDGs is defined as "Quality Education" [4], and this goal is known as SDG-4 or GG-4. The fifth goal of the SDGs is the goal which returns to the diversity of the gender, where this goal has multiple aims which mainly focus on the equality between the two genders. The fifth goal of the SDGs is defined as "Gender Equality" [5], and this goal is known as SDG-5 or GG-5. The goal which focuses on the water is the sixth goal of the SDGs. This goal aims to secure the clean water for all people over the world. The sixth goal is defined as "Clean Water" and "Sanitation" [6], and this goal is known as SDGs-6 or GG-6. The main environmental goal is the seventh SD goal which aims for securing the clean energy sources for all societies over the world. The seventh goal of the SDGs is defined as "Affordable" and "Clean Energy " [7], and it is known as SDG-7 or GG-7. The main economic goal of the SDGs is the eighth goal which has multiple aims such as securing the work for all aiming for growing the economy in all societies over the world. The eighth SD goal is defined as "Decent Work" and "Economic Growth" [8], and it is known as SDG-8 or GG-8. The goal which is related to the industry and manufacturing is the ninths goal which has multiple aims which mainly focus on the sustainable industry for all parts of the world. The ninth goal is defined as "Industry", "Innovation", and "Infrastructure" [9], and it is known as SDG-9 or GG-9. While the fourth SD goal focuses on gender equality, the tenth goal of the SDGs focuses on equality between and in different countries in the world. The tenth goal has multiple aims such as the equality between the developing countries and developed countries. The tenth SD goal is defined as "Reduce Inequality" [10], and it is known as SDG-10 or GG-10. The eleventh goal of the SDGs focuses on improving the suitability in the communities and cities where this can be achieved using multiple aims relate to multiple faces such as the houses and transportation. The eleventh goal is defined as "Sustainable Cities" and "Sustainable Communities" [11], and it is known as SDG-11 or GG-11.

**Fig. 1:**
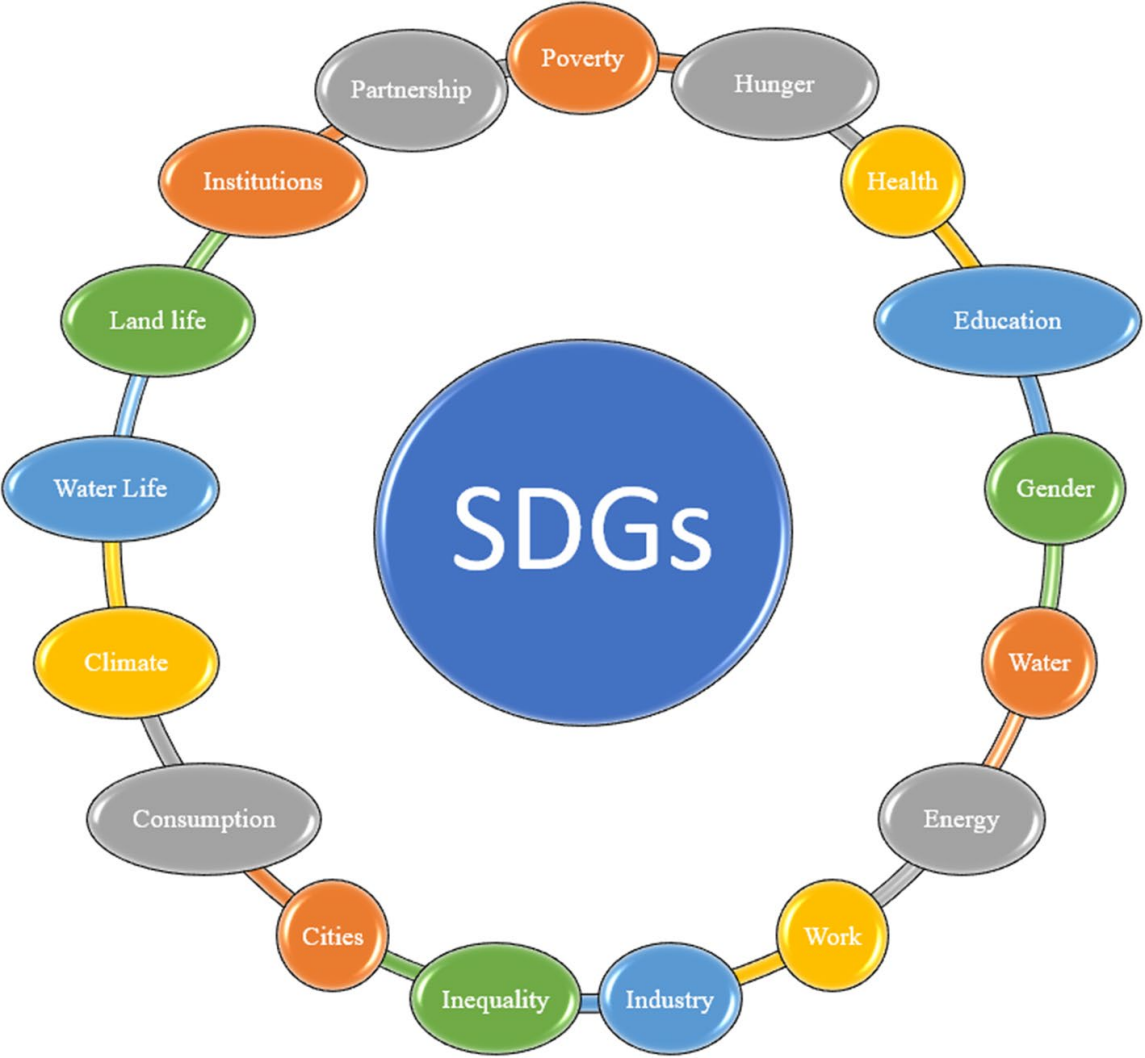
The SDGs

Another economic goal of the SDGs is the twelfth goal which mainly focuses on the responsible consuming of the resources from different faces such as the consuming of water and consuming of electricity. The twelfth goal is defined as "Responsible Consumption" and "Production" [12], and this goal is known as SDG-12 or GG-12. Because of the need to observing the climate changes and their effects on the world, the United Nation include the thirteenth goal which focuses on the climate. The thirteenth goal is defined as "Climate Action" [13], and this goal is known as SDG-13 or GG-13. The fourteenth and fifteenth SD goals focus on the life under water, especially in oceans, and the live on the land. The fourteenth goal is defined as "Life" below "Water" [14], while the fifteenth goal is defined as "Life" on "Land" [15]. The two SDGs are known as SDG-14, and SDG-15 or GG-14, and GG-15 respectively. Th sixteenth goal of the SDGs focuses on multiple effects, especially which are related to the output of the institutions, and peace. The sixteenth goal is defined as "Peace", "Justice", and "Strong Institutions" [16], and it is known as SDG-16 or GG-16. The last goal is one of the most important SDGs, which focuses on the collaboration for achieving the sustainable goals, and it is the main output of the United Nation. The seventieth goal of the SDGs goal is defined as "Partnerships for the Goals" [17], and it is known as SDG-17 or GG-17. In the Fig. 1, the full list of the sustainable development goals is illustrated. Each one of the goals has multiple indicators and aims for reaching or achieving it. As it can be seen from the SDGs list, the goals are complimentary to each other where achieving one goal may lead to another goal. For instance, if we apply the strategy of the using of renewable energy sources, we achieve one of the SDGs-7 targets, and in the same times we achieve one of the SDGs-12 indicators. Applying the sustainable development goals, completely or partially, in the developing countries and the low incoming countries can help with multiple problems in those countries. In this case study, we discuss the sustainability in an institution of one of the developing and low incoming country which is the Syrian Arab Republic. The study of the sustainability of the higher education systems are discussed in many literatures studies. For instance, the digital transformations effects were discussed [18]. Cotton, and Winter showed some practices of the sustainability in the higher education systems [19]. Yoo, and Jeon discussed the sustainability in the South Korean intuitions for some procedures [20]. Hossain et al. discussed some effects on the sustainability during the COVID-19 crisis by the employment of the bibliometrics methods [21]. Also, the bibliometrics methods are applied for the study of the sustainability for the police of the economy and environments [22], and for the libraries managements [23]. The relationship between the sustainability and the languages in the higher education learning are also discussed in multiple studies such as the study [24] for English language, and [25] for French language. In other sides, the effects of the virtual games in higher education business are discussed [26]. Sandanayake, Bouras, and Vrcelj showed the environmental results with the construction in Australia [27]. Bustamante, Martinovic, and Afflerbach showed the sustainability and some teaching effects in the higher education [28]. We discuss the sustainable development goals in Damascus University which is the largest institution in the Syrian Arab Republic. We study the plans which applied in the university for reaching lots of the sustainability goals in the last four years from 2018 to 2022 in addition to the beginning of 2023. In the second section of the case study, we talk about the main method applied in this study for retrieving Scopus and SciVal data for Damascus University for the SDGs of the university in the last four years. In the third section of the case study, we talk about the results of the methods which were applied in the same period. In the last section of the study, we illustrate the conclusion remarks, and the possible future procedures.

### Methods

The study of the procedures which any university applies for reaching the SDGs can be done by analysis of that procedures either from the most important results which resulted by the procedures, or by analysis the results using multiple techniques. In the second section we analyse the resulted useful aspects of applied procedures in Damascus University by illustrating the main results and discussed those results. Also, we analyse the results of the procedures by bibliometric study of the researches published in Scopus database and SciVal which return to the researchers in Damascus University. For this purpose, we first find the University profile in the database including all campuses or other name of the university. For instance we use the following search string "AF-ID ("Damascus University" 60072760) OR AF-ID ("Damascus University Faculties of Dentistry" 60072764) OR AF-ID ("University of Damascus School of Medicine" 60072761) ". Based on that search, we find the total number of the published documents by the researchers affiliated with Damascus University. After that, we apply the search including each one of the sustainable development goals collaborated wit the search string of Damascus University as full or whole institution. For instance, for finding the results of Damascus University for the SDG-15, we apply the following search string ""TITLE-ABS-KEY ((terrestrial OR land OR inland OR freshwater) AND (biodivers* OR {species richness} OR bioeconom* OR bio-econom* OR {biological production} OR deforest* OR desertif* OR {earth system} OR {ecological resilience} OR ecosystem* OR eco-system* OR {trophic cascade} OR {trophic level} OR {trophic web} OR {threatened species} OR {endangered species} OR {extinction risk} OR {extinction risks} OR poach* OR {wildlife product} OR {wildlife products} OR {wildlife traffic} OR {wildlife market} OR {wildlife markets} OR {wildlife trafficking} OR {invasive species} OR {alien species} OR {land uses} OR {land use} OR {land uses} OR {land degradation} OR {soil degradation} OR {LULUCF} OR *forest* OR {land conservation} OR wetland* OR mountain* OR dryland* OR {mountainous cover} OR {protected area} OR {protected areas} OR {REDD} OR {forest management} OR {silviculture} OR {timber harvest} OR {illegal logging} OR {slash-and-burn} OR {fire-fallow cultivation} OR {tree cover} OR {soil restoration} OR {land restoration} OR {drought} OR {sustainable land management} OR {mountain vegetation} OR {habitat restoration} OR {Red List species} OR {Red List Index} OR {extinction wave} OR {habitat fragmentation} OR {habitat loss} OR {Nagoya Protocol on Access to Genetic Resources} OR {genetic resources} OR {biological invasion} OR {biodiversity-inclusive} OR {forest stewardship council} OR {rainforest alliance} OR {forest certification} OR {forest auditing} OR {ecotourism} OR {community-based conservation} OR {community based conservation} OR {human-wildlife conflict})) AND AF-ID ("Damascus University" 60072760) OR AF-ID ("Damascus University Faculties of Dentistry" 60072764) OR AF-ID ("University of Damascus School of Medicine" 60072761)"." As we see from this search string, the search string includes the string returns to the fifteenth sustainable development goal in addition to Damascus University search string. The same procedure is applied for other SDGs. Based on the full searches, we find all bibliometrics data related to the sustainability results of the researchers affiliated with Damascus University where we analyse those results in the third section of the study. We deal with the main procedures which have been applied in Damascus University for reaching lots of the indicators of the sustainable development goals in the university. For more than four years, the University of Damascus has been adopting a policy of sustainable development in a large number of aspects related to the educational, societal, and research aspects. There was an urgent need to rely on the policy of sustainable development in these aspects, given the approach adopted by the university in responding to the call of the United Nations in the agenda of its General Assembly in the year 2015, in addition to the results emanating from the Syrian crisis, which negatively affected the educational process at the university. Multiple procedures were applied in Damascus university for the responding to the goals of sustainability. Firstly, in 2018, Damascus University launched a digital transformation policy in order to rely on the digital policy and replace the paper and print policy by the digital policy. Depending on the digital transformation policy, the first important thing that the university did at that time was to create a digital database for the certificates issued by it, by relying on an electronic sticker that is scanned electronically or writing the certificate number on the university’s website on the Internet. The certificates initially included master's and doctoral degrees, to be expanded later to include bachelor's degree certificates. Another important thing related to the digital transformation policy at the University of Damascus is the cancellation of the printed academic journals published by the university and replacing them with online journals only, which contributes to saving a huge amount of papers, which reflects positively on two of the goals of sustainable development. Previously, before 2018, the academic journals published by the University of Damascus relied on printing manuscripts in order to peer-reviewed, but starting in 2018, the university relied on the mechanism of open journal system (OJS) in peer-reviewing. In addition, and in order to reduce the use of paper, Damascus University began in 2022 to announce the results of exams electronically only through the official website of the university on the Internet, while before that it relied on announcing the results on paper and electronically in many of its faculties and institutes. Also, in the year 2018, and in order to respond to the goals of sustainable development, Damascus University called for the ideal use of renewable energies. Where the university initially signed a number of agreements with a number of research centers concerned with energy research in order to install a number of solar electric energy cells and solar energy cells related to heating matters on the roofs of the university buildings. One of these agreements related to energy is the agreement signed between the University of Damascus and the National Center for Energy Research in the Syrian Arab Republic. The previous agreement aimed at installing electric solar cells in the university, which contributed to reducing the electrical energy generated from fuel. Also, in the field of health, and in order to respond to the goals of sustainable development related to health, Damascus University has started to hold many events and conferences related to health awareness, starting in 2019. These events and conferences at the university increased after the spread of the new corona-virus epidemic in the Syrian Arab Republic. These conferences and events had their own impact on community health, and this was reflected in the research published on behalf of the University of Damascus in international databases such as Scopus. In the field of public health as well, the University of Damascus has purchased and installed a number of medical equipment related to public health in its various hospitals, namely: Al-Mowasat University Hospital, University Children's Hospital, University Obstetrics and Gynecology Hospital, Al-Assad University Hospital, University Dermatology Hospital, Al-Bayrouni University Hospital, and the Institute dentistry at the university. As a result of the Syrian crisis, which greatly affected the electricity sector and its generation, Damascus University called on many occasions for the rationalization of electricity consumption in the various university buildings in its various branches, and this affects one of the goals of sustainable development and is an important indicator in it. The University of Damascus has also called on several occasions to rationalize water consumption in all university buildings and in its various branches. The university has been doing this even before the emergence of the Sustainable Development Goals in 2015. This reflects positively on one of the goals of sustainable development and is an important indicator in that goal. The most important response to the sustainable development goals by the University of Damascus was in the environmental field. The university interest in expanding the green area has increased in relation to the total area in the university, and the university has encouraged all its faculties and all its branches to take advantage of the fertile soil in the lands belonging to the University of Damascus in increasing the green area. A number of things helped in this, the most important of which is the presence of sufficient factors for agriculture in the lands belonging to the University of Damascus, and the desire of a large number of professors, teachers and university employees to increase the green area in it. In addition to that, in the field of environment, botanic gardens are built in the faculties of science of Damascus University, which contributes to the diversity of plants present in them and is reflected in many indicators within the goals of sustainable development. Also, in the same field, the university encouraged all of the faculties members, the managers, the employees, and the students for reducing the emission of the carbon dioxide, and other greenhouse gases.

## Results and discussion

In this section, we analyse the main output resulted from applying the SDGs procedures in Damascus University. In addition, we analyse the bibliometrics data rederived from the databases. Firstly, we deal with the main procedures which have been applied in Damascus University for reaching lots of the indicators of the sustainable development goals in the university. For more than four years, the University of Damascus has been adopting a policy of sustainable development in a large number of aspects related to the educational, societal, and research aspects. There was an urgent need to rely on the policy of sustainable development in these aspects, given the approach adopted by the university in responding to the call of the United Nations in the agenda of its General Assembly in the year 2015, in addition to the results emanating from the Syrian crisis, which negatively affected the educational process at the university. Multiple procedures were applied in Damascus university for the responding to the goals of sustainability. Firstly, in 2018, Damascus University launched a digital transformation policy in order to rely on the digital policy and replace the paper and print policy by the digital policy. Depending on the digital transformation policy, the first important thing that the university did at that time was to create a digital database for the certificates issued by it, by relying on an electronic sticker that is scanned electronically or writing the certificate number on the university’s website on the Internet. The certificates initially included master's and doctoral degrees, to be expanded later to include bachelor's degree certificates. Another important thing related to the digital transformation policy at the University of Damascus is the cancellation of the printed academic journals published by the university and replacing them with online journals only, which contributes to saving a huge amount of papers, which reflects positively on two of the goals of sustainable development. Previously, before 2018, the academic journals published by the University of Damascus relied on printing manuscripts in order to peer-reviewed, but starting in 2018, the university relied on the mechanism of open journal system (OJS) in peer-reviewing. In addition, and in order to reduce the use of paper, Damascus University began in 2022 to announce the results of exams electronically only through the official website of the university on the Internet, while before that it relied on announcing the results on paper and electronically in many of its faculties and institutes.

**Fig. 2:**
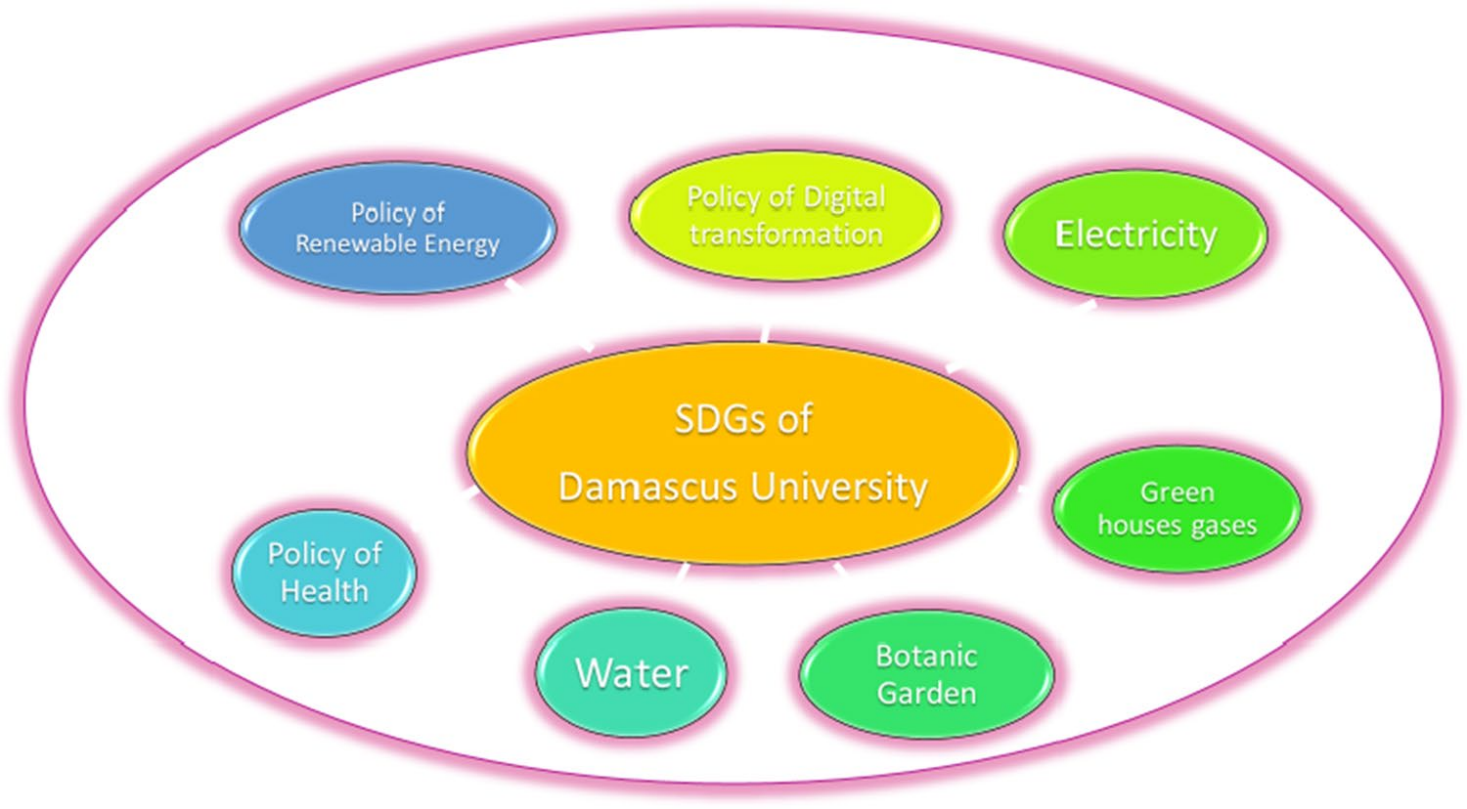
The main procedures in Damascus University for responding to the SDGs programs

Also, in the year 2018, and in order to respond to the goals of sustainable development, Damascus University called for the ideal use of renewable energies. Where the university initially signed a number of agreements with a number of research centers concerned with energy research in order to install a number of solar electric energy cells and solar energy cells related to heating matters on the roofs of the university buildings. One of these agreements related to energy is the agreement signed between the University of Damascus and the National Center for Energy Research in the Syrian Arab Republic. The previous agreement aimed at installing electric solar cells in the university, which contributed to reducing the electrical energy generated from fuel. Also, in the field of health, and in order to respond to the goals of sustainable development related to health, Damascus University has started to hold many events and conferences related to health awareness, starting in 2019. These events and conferences at the university increased after the spread of the new corona-virus epidemic in the Syrian Arab Republic. These conferences and events had their own impact on community health, and this was reflected in the research published on behalf of the University of Damascus in international databases such as Scopus. In the field of public health as well, the University of Damascus has purchased and installed a number of medical equipment related to public health in its various hospitals, namely: Al-Mowasat University Hospital, University Children's Hospital, University Obstetrics and Gynecology Hospital, Al-Assad University Hospital, University Dermatology Hospital, Al-Bayrouni University Hospital, and the Institute dentistry at the university. As a result of the Syrian crisis, which greatly affected the electricity sector and its generation, Damascus University called on many occasions for the rationalization of electricity consumption in the various university buildings in its various branches, and this affects one of the goals of sustainable development and is an important indicator in it. The University of Damascus has also called on several occasions to rationalize water consumption in all university buildings and in its various branches. The university has been doing this even before the emergence of the Sustainable Development Goals in 2015. This reflects positively on one of the goals of sustainable development and is an important indicator in that goal. The most important response to the sustainable development goals by the University of Damascus was in the environmental field. The university interest in expanding the green area has increased in relation to the total area in the university, and the university has encouraged all its faculties and all its branches to take advantage of the fertile soil in the lands belonging to the University of Damascus in increasing the green area. A number of things helped in this, the most important of which is the presence of sufficient factors for agriculture in the lands belonging to the University of Damascus, and the desire of a large number of professors, teachers and university employees to increase the green area in it. In addition to that, in the field of environment, botanic gardens are built in the faculties of science of Damascus University, which contributes to the diversity of plants present in them and is reflected in many indicators within the goals of sustainable development. Also, in the same field, the university encouraged all of the faculties members, the managers, the employees, and the students for reducing the emission of the carbon dioxide, and other greenhouse gases. In the Fig. 2, we illustrate the main indicators which Damascus University apply for the reaching to some of the sustainable development goals.

In this section, we talk about the main significant results of responding of Damascus University to the SDGs. Firstly, we start by some important information about Damascus University. Damascus University is the largest university in the Syrian Arab Republic and its built returns to the beginning of the Twentieth century. Currently, the university has more than 200,000 students over all parts of the university. Damascus University grants a number of programs, starting from a bachelor's degree or its equivalent, up to a doctorate degree. The university has its valuable Motto which is "My Lord, increase me in knowledge". The main targets of the university are the scientific research and the educational aspects. Since its foundation, the university has put all possibilities in order to achieve its previous targets.

In the year 2018, and three years after the emergence of the sustainable development goals in 2015, the university sought to achieve a number of indicators related to some of the sustainable development goals, starting from environmental indicators to societal indicators. One of the main goals in reaching the sustainable development goals was to benefit from them in developing the educational, research and knowledge process at Damascus University. There are multiple departments and faculties in Damascus University focus on the sustainability goals. Those departments and faculties are illustrated in the Table 1, which includes the main institution, the SDGs faculties, and the SDGs departments. Although, there are other faculties and departments found in Damascus University can apply effective procedures for the reaching to the SDGs, those faculties do not apply actual procedures for the reaching to the substantiality goals. For example, Faculty of economic, Faculty of Civil Engineering, Faculty of Education, Higher Institute of Administrative Development, and Faculty of Architecture. Here we refer to the existence of three other branches of the University of Damascus in three other Syrian governorates, which are Quneitra, As-Suwayda, and Daraa. All of these branches include a number of faculties similar to that shown in the Table 1, in addition to the main branch in Damascus.

**Table 1:** The main faculties and departments in Damascus University which focus on the SDGs

Damascus University							
Faculty of Sciences			Faculty of Mechanical and Electrical Engineering		Faculty of Agriculture	Faculty of Humanities	Faculty of Medicine
Department of Physics	Department of Chemistry	Department of Environment	Department of Energy	Department of Renewable Energy	All departments of the faculty	Department of Geography	All departments of the faculty

All of these four campuses work on some indicators of the SDGs, for instance, in the Faculty of Sciences in As-Suwayda, which is called the fourth Faculty of Sciences of Damascus University, there is a botanic garden and there are lots of work for increasing the plants and trees in that campus. There are lots of the significant results resulted from applying sustainability procedures by Damascus University. The first important result of applying the sustainability procedures returns to generating the energy based on the renewable energy sources, where Damascus University work in two directions for this target. The first direction is the use of the electrical solar cells for generating the electricity from the main energy source of the earth which is the sun. The other direction is the use of the heating solar source for the heating of water. These two procedures have affected the ratio of the total consumed electricity in Damascus University. In the Table 2, we illustrated the total amount of consumed electrical energy in the last year, and the total amount of the electrical energy generated by the renewable energy or its equivalence. The generated energy includes the electrical energy generated by the electrical solar cells, in addition to the equivalent electrical energy generated by the solar thermal cells. The main solar electrical cells in Damascus University are mounted on the roofs of the Faculty of Mechanical and Electrical Engineering, while the solar thermal cells are distributed over all parts of the university. As we see from the Table 2, the electrical energy which was generated from the solar electrical cells or photovoltaic cells and its equiveillance represents about 11% of the total consumed electric energy in all parts of the building of Damascus University. This important thing has its reflections on three sustainable development goals which are SDG-7, SDG-12, AND SDG-13. The first reflection on the SDG-7 returns to the using the clean energy sources based on the photovoltaic cells and solar thermal cell. The reflection on the SDG-12 returns to the reducing of the consuming of the electricity, while the reflection on the SDG-13 is due to the reduction of the amount of secondary emissions resulting from the traditional generation of electricity by means of fuel or gas. Also, in the Table 2, we illustrate the total amount of the consumed water in the last year in cubic meters units, and the approximated total amount of the emission of greenhouses gases in the kilogram carbon dioxide equivalence units which include the total amount of the emission of the greenhouses gases. The total amount of water represented in the Table 2 includes the humans, animals, and the using of water for plants, while the total amount of the carbon emission includes the humans, electrical generation, and animals. If we go back to the amount of water consumed at Damascus University two years ago, we find that it is one and a half times higher than last year. Besides, if we go back to the amount of carbon emissions in the university before 2018, we find that it is estimated at about 13,000 kg carbon dioxide equivalence per minute which means that the total amount of the carbon emission has been reduced to more than its half value. The previous procedures related to the water and the carbon emission reflect on the SDG-6, SDG-12, and SDG-13. In the health field, Damascus University has taken strides in installing a number of devices related to public health in its university hospitals. In addition to installing a large number of water purifiers in a large number of university buildings. These aspects reflect mainly on the SDG-3. In the goals related to the environmental procedures, Damascus University released the policy related to the increasing of the plants and trees in all parts of the buildings of the university. In the Table 3, we have included the land plan of the total area of Damascus University in all its buildings in square meters units, in addition to the cultivated area or the green area in the last year.

**Table 2:** The total amount of the consumed water, the total amount of the carbon emission, the total amount of the consumed energy, and the total amount of the energy generated by renewable sources of energy in Damascus University

The total amount of the consumed water (cubic meter)	The total amount of carbon emissions (kgCO_2_e per minute)	The total consumed electrical energy (kWh)	The total electric energy generated by renewable sources (kWh)
653,850	5793	27,870,336	3,065,737

**Table 3:** The green areas in Damascus University and the total plan area of all university buildings

The land plan area of Damascus University (square meter)	The green area (square meter)
152,600	233,692

As we can see from the Table 3, the green area of Damascus University represents about 65.3% of the total land plan of the university.

**Fig. 3:**
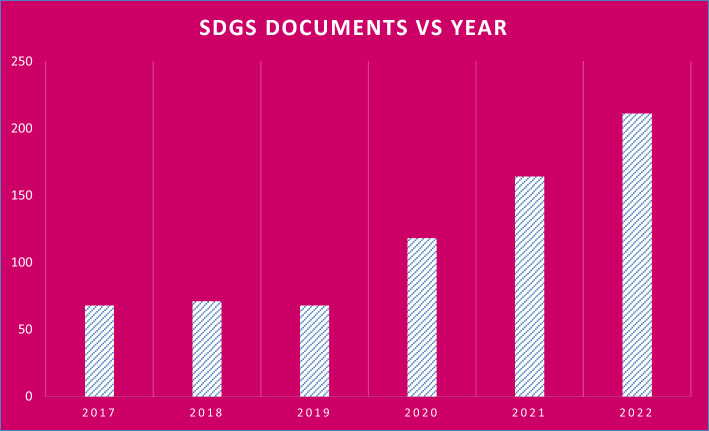
The total SDGs documents published by researchers affiliated with Damascus University using Scopus and SciVal data

This significant aspect reflects mainly on the SDG-13. Finally, for the procedures related to the policy of digital transformation which the university focuses on, we see that the reducing of the consumed water has its reflection on the environmental, and educational goals of the SDGs. This aspect reflects mainly on the SDG-4, and SDG-13. In addition, this aspect has partial reflection on the SDG-8, and the SDG-12 because it has economic effects. Also, we analyses the bibliometrics data which returns to the SDGs of Damascus University. As we mentioned in the second, we found the Scopus data by applying two types of the search strings. Herein, we discuss the results of the searches. First, we illustrate the total number of the published documents of the SDGs, which Indexed and abstracted in the Scopus database, during the period from 2017 to 2022 and published by researches from Damascus university. We plotted these results in the Fig. 3 which includes the published SDGs documents versus year of the publishing. As we see from the Fig. 3, the publishing of the SDGs researches by the Damascus University researchers increases with the time. We see that total number of the published documents in the sixteen suitable development goals reaches to 211 published documents. Also, we represent the SDGs published documents by the Researchers from Damascus University in 2022 because that year was the year with highest number of the published SDGs documents which indexed and abstracted in the Scopus database.

**Fig. 4:**
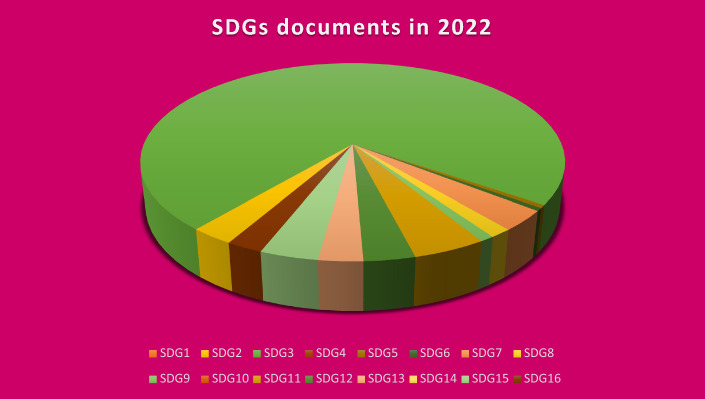
The SDGs documents published by researchers from Damascus University in 2022 for the sixteen sustainable development goals

As we can see from the Fig. 4, the sustainable development goal with the highest published documents is the SDG-3 which returns to the good health targets. We see that we have the 155 published documents about the good health SDG in 2022 while the total number of the published documents by the researchers from Damascus University is 211 in that year. This means that the SDG-3 represents more than a half of the total published documents about the sustainable development goals in Damascus University. aspect is resulted from the publishing in the Faculty of Medicine in Damascus University and the encouraging of the health care, especially after the new coronavirus disease crisis which also affects this point.

**Fig. 5:**
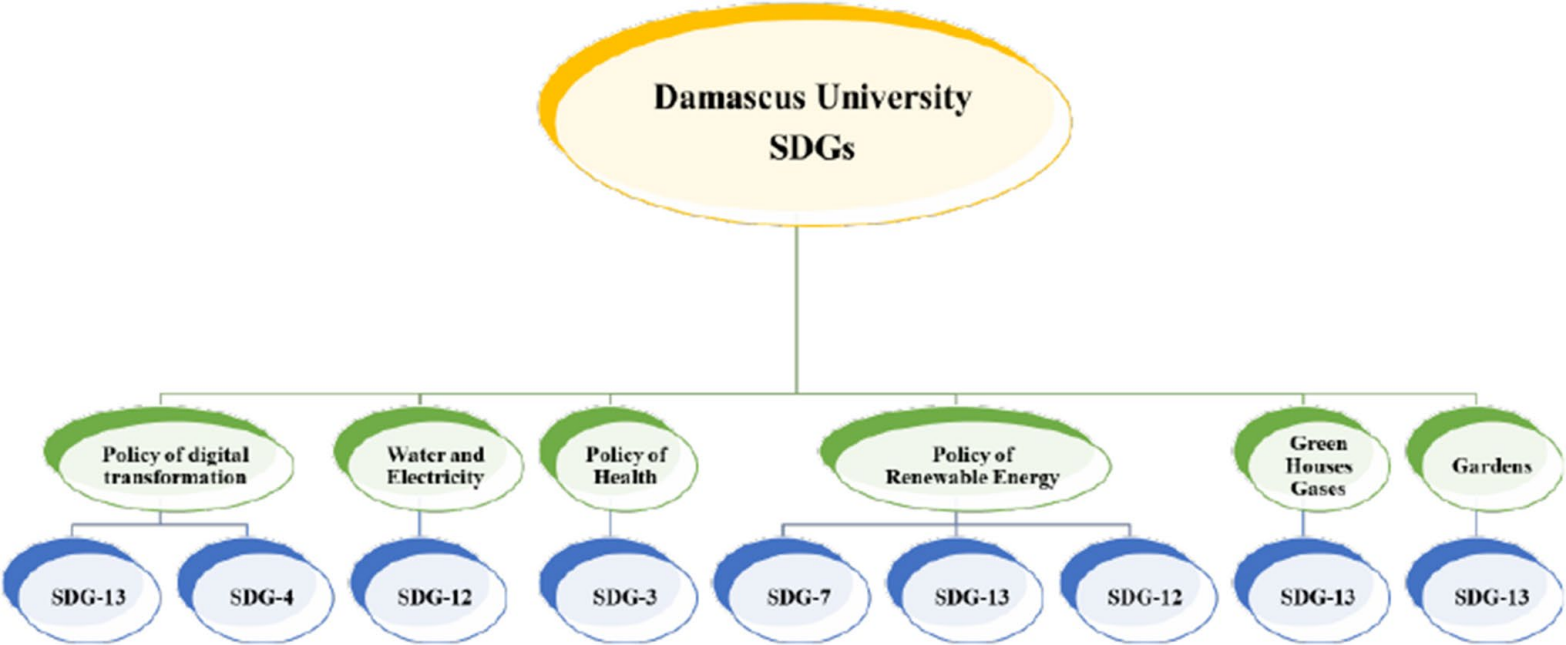
The suitable development goals achieving in Damascus University

Finally, in the Fig. 5, we represented the current achievements of the sustainable development goals in Damascus University and which they remain in progress. As it can be seen from the Fig. 5, the policy of the digital transformation affects the SDG-13, and the SDG-4. The procedures related to the saving of water and the electricity consuming mainly affect on the reaching the SDG-12. The policy of public health and supporting the students in the faculty of medicine and the university hospital for reaching the good health of the societies in the country affects the SDG-3. The using of renewable energies for generating the electric current has multiple effects on the reaching the suitable developments goals where it affects mainly on the SDG-7, the SDG-13, and the SDG-12. Also, the policy of the increasing of the green areas in the buildings of the university affects mainly on the SDG-13, and the same thing is for the policy of reducing the green houses gases which are generated from the building of the university.

## Conclusions

The aim of this research is to present the policies pursued by the University of Damascus in reaching the sustainable development goals launched by the United Nations in its General Assembly in 2015. Damascus University was chosen for several reasons, the most important of which is that it is the largest university in the Syrian Arab Republic, which is an example of low-income countries and an example of developing countries. We stated that the university started implementing sustainable development policies in 2018 in many aspects. In 2018, the university began with a policy of digital transformation that led to a significant reduction in the use of paper and paper publications, by switching to the use of electronic certificates for university graduates, in addition to the use of the open journals system in the academic journals published by the university. Also, in the same year, the university began to implement the policy of renewable energies effectively by using solar electric panels to generate electricity and solar thermal cells to heat water. In the year 2022, this policy led to the generation of clean energy estimated at about eleven percent of the electrical energy used by the university in that year. We found that the university called for a policy of rationalizing the use of water in various aspects and for a policy of reducing carbon emissions in university buildings. We also found that the application of some health-related policies had the greatest impact through research published on behalf of the University of Damascus in international databases such as Scopus. In addition, we found that one of the most important results emanating from the university's policy in reaching the goals of sustainable development is the increase in the percentage of cultivated area or green space, which approached more than 63 percent of the total projected area in all university buildings. Based on the bibliometrics analysis of the SDGs based on the Scopus and SciVal data, we found that the SDG-3 targets were the most publishing documents by the Syrian researchers affiliated with Damascus University and represented more than 55% of the total published documents about the SDGs in 2022.

As a result of implementing the sustainability policy at the University of Damascus, the university was able to reach indicators related to a good number of sustainable development goals, namely: SDG-3, SDG-4, SDG-7, SDG-12, and SDG-13. Also, the university reached partially to the SDG-8. As the sustainability strategy of Damascus University states that the university remains for reaching to other indicators of the SDGs. Damascus University is following up its procedures to achieve sustainable and effective development in the Syrian society. As an example of the future ways in which the university follows up its procedures to apply the policy of sustainable development in the field of education, which was severely affected during the Syrian crisis [29], especially what was affected the school in the Syrian Arab Republic. And also start adopting a recycling policy in all faculties of the university in all its four branches in the Syrian Arab Republic. In this context, the university began to take steps towards directing its students towards a recycling policy, in addition to recommendations to start installing recycling garbage containers.

## Data Availability

All data generated or analysed during this study are included in this published article [and its supplementary information files].
